# Mechanisms of sepsis‐induced immunosuppression and immunological modification therapies for sepsis

**DOI:** 10.1002/ags3.12194

**Published:** 2018-07-24

**Authors:** Satoshi Ono, Hironori Tsujimoto, Shuichi Hiraki, Suefumi Aosasa

**Affiliations:** ^1^ Division of Critical Care Medicine Tokyo Medical University Hachioji Medical Center Tokyo Japan; ^2^ Department of Surgery National Defense Medical College Saitama Japan

**Keywords:** anti‐programmed cell death 1, interleukin‐10, interleukin‐7, regulatory T cell, sepsis

## Abstract

Surgical injury can be a life‐threatening complication, not only due to the injury itself, but also due to immune responses to the injury and subsequent development of infections, which readily result in sepsis. Sepsis remains the leading cause of death in most intensive care units. Unfavorable outcomes of several high‐profile trials in the treatment of sepsis have led researchers to state that sepsis studies need a new direction. The immune response that occurs during sepsis is characterized by a cytokine‐mediated hyper‐inflammatory phase, which most patients survive, and a subsequent immunosuppressive phase. Therefore, therapies that improve host immunity might increase the survival of patients with sepsis. Many mechanisms are responsible for sepsis‐induced immunosuppression, including apoptosis of immune cells, increased regulatory T cells and expression of programmed cell death 1 on CD4^+^ T cells, and cellular exhaustion. Immunomodulatory molecules that were recently identified include interleukin‐7, interleukin‐15, and anti‐programmed cell death 1. Recent studies suggest that immunoadjuvant therapy is the next major advance in sepsis treatment.

## 1 INTRODUCTION

Surgical injury can be a life‐threatening complication, not only due to the injury itself, but also due to immune responses to the injury and the subsequent development of infections with or without associated organ dysfunction. Patients who undergo major surgery for gastrointestinal cancer are at high risk of postoperative infection. Postoperative infectious complications may be caused by postoperative immunosuppression associated with dysregulation of cytokine production. Suppression of cellular immunity is a host response to surgical stress that readily leads to sepsis. Therefore, improving the immune dysfunction of postoperative patients might play a crucial role in preventing severe complications following major surgery.

Sepsis is a common and frequently fatal clinical condition occurring in critically ill patients. Septic patients frequently present with fever, shock, and respiratory failure as a result of an uncontrolled proinflammatory response that has been termed systemic inflammatory response syndrome (SIRS).^1^ Definitions of sepsis were last revised in 1992. These definitions were focused on the SIRS of the host to infection. However, the validity of SIRS as an indicator of sepsis pathobiology has remained controversial. Sepsis is now recognized to involve the early activation of both pro‐ and anti‐inflammatory responses. The current use of ≥2 SIRS criteria to identify sepsis was unanimously considered by the task force to be unhelpful. The SIRS criteria do not necessarily indicate a dysregulated life‐threatening response. Thus, the public is in need of an understandable definition of sepsis. Sepsis is defined as life‐threatening organ dysfunction caused by a dysregulated host response to infection. Organ dysfunction was identified as an acute change in total Sequential Organ Failure Assessment score^2^ (SOFA) of ≥2 as a consequence of the infection (Table 1).

**Table 1:** New definitions of sepsis

SOFA score	1	2	3	4
Respiration				
PaO_2_/FiO_2_, mm Hg with respiratory support	<400	<300	<200	<100
Coagulation				
Platelets × 10^3^/mm^3^	<150	<100	<50	<20
Liver				
Bilirubin, mg/dL	1.2‐1.9	2.0‐5.9	6.0‐11.9	>12.0
Cardiovascular				
Hypotension	MAP <70 mm Hg	Dopamine ≦5	Dopamine >5 Norepinephrine ≦0.1	Dopamine >15 Norepinephrine <0.1
Central nervous system				
Glasgow Coma Scale	13‐14	10˜12	6˜9	<6
Renal				
Creatinine, mg/dL or urine output	1.2‐1.9	2.0‐3.4	3.5‐4.9 <500 mL/d	>5.0 <200 mL/d

Sepsis is defined as life‐threatening organ dysfunction caused by a dysregulated host response to infection.

Organ dysfunction can be identified as an acute change in total SOFA score of ≧2 points consequent to infection.

MAP, mean arterial pressure; SOFA, Sequential Organ Failure Assessment.

## 2 MECHANISM OF SEPSIS‐INDUCED IMMUNOSUPPRESSION

This initial immune recognition response is mediated by pathogen‐associated molecular patterns and damage‐associated molecular patterns originating from bacterial or fungal organisms that blind pattern recognition receptors expressed on innate immune cells.^3^ The activation of pattern recognition receptors results in the production of numerous proinflammatory cytokines, including tumor necrosis factor (TNF)‐α, interleukin (IL)‐1β, IL‐6, IL‐8, and interferon (IFN)‐γ and anti‐inflammatory cytokines that induce excessive hyper‐inflammatory responses and counter‐responses. These responses include chemotaxis of leukocytes to sites of infection/inflammation, vascular endothelial injury with capillary leak, and activation of the coagulation system.^4^

Until recently, most research on sepsis was focused on blocking the initial hyper‐inflammatory response. Initially, the proinflammatory response was believed to be the major cause of mortality in patients with sepsis and was frequently targeted for therapeutic intervention.^5^ However, efforts to improve outcomes by targeting proinflammatory cytokines and mediators, such as TNF and IL‐1β antagonists, endotoxin antagonists, Toll‐like receptor (TLR) blockers, and platelet activating factor inhibitors, have been unsuccessful.^6^

**Figure 1:**
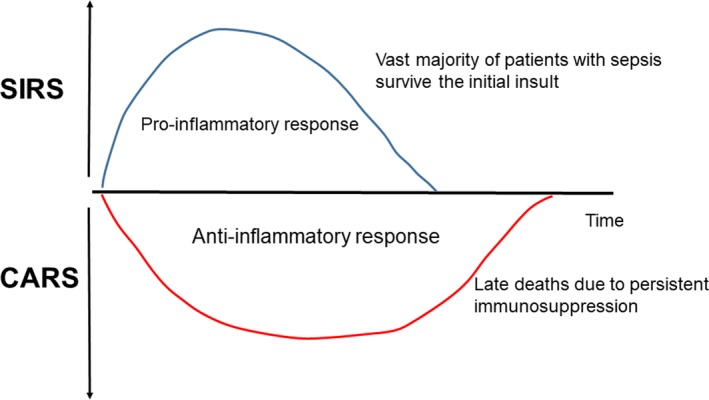
Host immune response in sepsis. Activation of both proinflammatory and anti‐inflammatory immune responses occur promptly after sepsis onset. Cells of the innate immune system release high levels of proinflammatory cytokines. Most patients recover from the hyper‐inflammatory “cytokine storm” response and survive the infection. If sepsis persists, the immune system fails and an immunosuppressive state occurs in such patients. CARS, compensatory anti‐inflammatory response syndrome; SIRS, systemic inflammatory response syndrome

This profound proinflammatory state, which occurs during the early onset of sepsis, is rapidly counterbalanced by an anti‐inflammatory response, which may adversely affect immune functions.^7^ This was initially referred to as compensatory anti‐inflammatory response syndrome.^8^ The vast majority of patients with sepsis survive the initial insult. Sepsis‐induced immunosuppression is increasingly recognized as the overriding immune dysfunction in these vulnerable patients^7^ (Figure 1). Immunosuppression in sepsis thus provides a novel understanding of the disorder as well as a new therapeutic approach.^9^

### 2.1 Host immune response in sepsis

Recent studies show that the activation of both proinflammatory and anti‐inflammatory immune responses occurs promptly after the onset of sepsis.^10^ Cells of the innate immune system, including monocytes and neutrophils, release high levels of proinflammatory cytokines. The rapid deaths of patients with sepsis are typically owing to a hyper‐inflammatory “cytokine storm” response. If sepsis persists, the failure of crucial elements of both the innate and the adaptive immune system occurs, such that patients enter a marked immunosuppressive state.^10^ We have shown that patients who die of sepsis have marked immunosuppression^11^ (Figure 1). Deaths are the result of an inability of patients to clear their primary infections, as well as the development of secondary infections.

Sepsis can be considered to represent a race between the pathogens and the host immune response; pathogens seek an advantage by incapacitating various aspects of host defenses. For example, sepsis induces the apoptotic deletion of immune effector cells, suppresses the expression of major histocompatibility complex class II molecules, increases the expression of negative costimulatory molecules, increases anti‐inflammatory cytokines, and increases the numbers of regulatory T cells and myeloid‐derived suppressor cells.^11^

### 2.2 Apoptosis and immunosuppression

Apoptosis is an irreversible reaction in which the immune system maintains homeostasis by eliminating activated cells.^12^ Central to apoptosis are caspases, which are cysteine proteases that degrade cellular proteins and nuclear factor‐kappa B (NF‐κB), a transcription factor that activates the transcription of both proapoptotic and prosurvival genes. Whereas hyper‐inflammatory responses of sepsis require NF‐κB for the production of proinflammatory cytokines and the activation by caspase cleavage, both NF‐κB and caspases concurrently induce the apoptosis of immune cells.^3^ Consistent with this, a concurrent apoptotic response has been shown to be present in sepsis in association with the proinflammatory response.^13^

**Figure 2:**
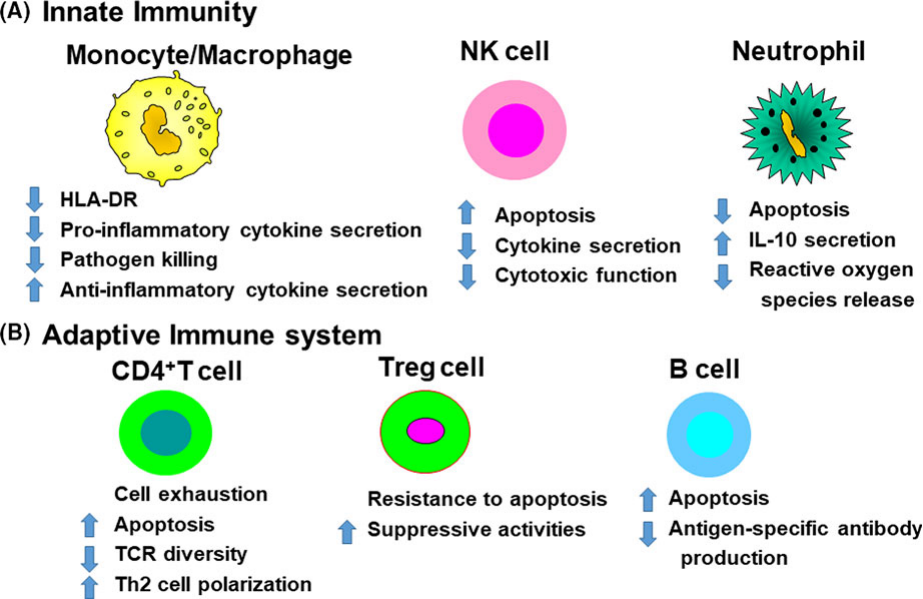
Sepsis‐induced immunosuppression. Apoptosis causes the marked deletion of immune cells, including natural killer (NK) cells, CD4^+^ T cells and CD8^+^ T cells, and B cells, in various organs of patients dying of sepsis, leading to immunosuppression. Decreased human leukocyte antigen (HLA)‐DR expression on antigen‐presenting cells, including monocytes and macrophages, is a hallmark of sepsis, which may impair optimal presentation of microbial antigens to T cells. IL‐10, interleukin 10; TCR, T cell receptor

Although the deletion of adaptive immune cells is recognized as an important part of the pathology of sepsis, the mechanisms responsible for this are not fully understood.^14^ Apoptosis causes the marked deletion of immune cells, including natural killer (NK) cells, CD4^+^ and CD8^+^ T cells, B cells, and dendritic cells (DC), in various organs of patients dying of sepsis, leading to immunosuppression (Figure 2). Apoptosis of immune cells occurs in lymphoid tissues and gut‐associated lymphoid tissues.^15^ The loss of intestinal intraepithelial and lamina propria lymphocytes might facilitate bacterial translocation into the systemic circulation, thereby perpetuating the systemic inflammatory response and predisposing to secondary infections. The detrimental effects of apoptosis are not only associated with the severe loss of immune cells but also with the effect that apoptotic cell uptake has on surviving immune cells.^16^ The uptake of apoptotic cells by monocytes, macrophages, and DC results in immune tolerance by inducing anergy or a T helper 2 cell‐associated immune phenotype with increased IL‐10 production.^17^

### 2.3 Monocytes and macrophages

We have already reported that patients with sepsis have monocytes with a decreased capacity to release proinflammatory cytokines in response to endotoxin.^18^ This finding is consistent with the phenomenon of endotoxin tolerance. Monocytes from patients with sepsis show a decreased ability to release proinflammatory cytokines, such as TNF, IL‐1, IL‐6, and IL‐12, whereas their ability to release anti‐inflammatory mediators, such as IL‐1 receptor antagonist and IL‐10, is either unimpaired or enhanced^19^ (Figure 2). In clinical studies, the magnitude and the persistent nature of this refractory state are associated with increased mortality. Two major consequences of endotoxin tolerance on monocytes are an increase in the release of immunosuppressive mediators, such as IL‐10, and a decrease in antigen presentation as a result of the reduced expression of human leukocyte antigen (HLA)‐DR; both are associated with a worse outcome of sepsis.^20^, ^21^ The continued release of IL‐10 might contribute to sepsis‐induced immunosuppression and thus might augment the susceptibility to secondary microbial infections.^22^ IL‐10 is produced by Treg cells and Th2‐type cells and suppresses the Th1 response, further potentiating an anti‐inflammatory environment.^23^ We have reported that blocking IL‐10 can reverse sepsis‐induced immunosuppression and improve survival in a mouse model of sepsis.^24^

Low levels of monocyte human leukocyte antigen (HLA)‐DR expression function as a surrogate marker of monocyte unresponsiveness.^25^ We previously reported that expression of the human leukocyte antigen HLA‐DR by peripheral blood monocytes was decreased in septic patients, particularly in patients with septic shock or severe sepsis.^26^ Several studies showed an association of reduced monocyte HLA‐DR expression with impaired monocyte function. These data show that monocyte unresponsiveness and immunosuppression independently contribute to the increased risk of adverse events in sepsis.

### 2.4 Natural killer cells

Natural killer cells are the principle producers of IFN‐γ during bacterial sepsis.^27^ These cells produce a large amount of IFN‐γ when stimulated with IL‐12 or IL‐18, both of which are produced by monocyte lineage cells activated by bacterial pathogens, such as endotoxin. IFN‐γ is the main activator of macrophages during sepsis, and NK cells have been shown to be major producers of IFN‐γ in polymicrobial sepsis; however, the role of NK cells during bacterial septic challenges remains largely undefined. We recently showed that the IFN‐γ‐producing capacity of human peripheral blood mononuclear cells (PBMC) is severely impaired in both postoperative patients after elective surgery and in septic patients.^28^ Impaired IFN‐γ production in response to lipopolysaccharide (LPS) has been reported in NK cells from patients with sepsis. Other studies indicated that sepsis affects the number of circulating NK cells, which is markedly decreased in patients with sepsis, and a low number of NK cells is associated with increased mortality. Furthermore, a decrease in the cytotoxic function of NK cells and cytokine secretion occurs during sepsis (Figure 2). NK cells in human PBMC are severely impaired by surgical stress to a greater extent than T cells or B cells. A recent study showed that NK cells are affected by surgical stress more severely than T cells or B cells.^29^

### 2.5 T cells

In sepsis, T cells become hypo‐responsive in terms of proliferation and turn toward a type 2 profile with an increased production of IL‐4 and IL‐10, and suppression of IL‐12 and IFN‐γ. We previously reported that serum IFN‐γ levels and IFN‐γ production by PBMC were significantly decreased in patients with sepsis compared with healthy volunteers.^28^, ^30^

In the late 1990s, Sakaguchi et al^31^ showed for the first time that the suppression mediated by CD4^+^ T cells appeared to result from the function of a small subset of T cells that expressed CD4^+^CD25^+^. These CD4^+^CD25^+^ T cells were reported to act on T cells through a cell‐contact mechanism involving cytotoxic T‐lymphocyte antigen,^32^ and are also thought to produce IL‐10 and transforming growth factor (TGF)‐β, and to suppress IFN‐γ production.^32^ Thereafter, Forkhead box protein 3 (Foxp3) was found to be expressed in CD4^+^CD25^+^ T cells, and these cells were subsequently named Treg cells. Treg cells are central to the maintenance of immunological homeostasis and tolerance.^33^, ^34^ In septic patients, the percentages of circulating Treg cells are markedly increased, which presumably contributes to the occurrence of sepsis‐induced immunosuppression.^35^ Our data showed that the total CD4^+^ T‐cell count and the percentage of CD4^+^ T cells in lymphocytes were significantly lower in patients with septic shock than in patients without septic shock.^36^ The percentage of Treg cells in the CD4^+^ T‐cell population, and serum IL‐10 and IL‐6 levels were significantly higher in patients with septic shock than in patients without septic shock. These clinical data indicate that IL‐10 may contribute to the increased percentage of Treg cells among the CD4^+^ T‐cell population under septic conditions, thus contributing to the immunosuppressive state associated with refractory sepsis. An increased percentage of circulating Treg cells has been described in patients with septic shock. This increase was observed immediately after the onset of sepsis but persisted only in those patients who subsequently died.^35^ These results indicated that this relative increase was probably caused by a decrease in effector T‐cell numbers. Recent studies indicated that an increased number of Treg cells is deleterious in sepsis and is associated with decreased effector T‐cell proliferation and function.^37^ Furthermore, Treg cells can also suppress innate immune cells. Treg cells inhibit both monocyte and neutrophil function,^38^ and induce an NK cell‐dependent endotoxin tolerance‐like phenomenon that is characterized by the decreased production of IFN‐γ.^39^ There is a large amount of evidence that patients with sepsis have increased numbers of Treg cells, which, by acting both on innate and adaptive immune cells, impair immunity and contribute to septic mortality.

T‐cell exhaustion has been shown in patients with sepsis. The prolonged duration of sepsis is characterized by high antigen load and high levels of proinflammatory and anti‐inflammatory cytokines, which induces T‐cell exhaustion. Boomer et al^11^ reported that spleens that were obtained rapidly after the death of patients with sepsis showed evidence that is highly consistent with the occurrence of T‐cell exhaustion; profound suppression of the production of IFN‐γ by stimulated T cells; increased expression of programmed cell death‐1 (PD‐1) on CD4^+^ T cells and programmed cell death 1 ligand 1 (PD‐L1) on macrophages. An association between T‐cell exhaustion and mortality in sepsis was provided by studies showing that the increased expression of PD‐1 in circulating T cells from patients with sepsis correlated with decreased T‐cell proliferative capacity and mortality.^40^

## 3 IMMUNOLOGICAL MODIFICATION THERAPIES

### 3.1 Immunostimulatory therapies in sepsis

**Figure 3:**
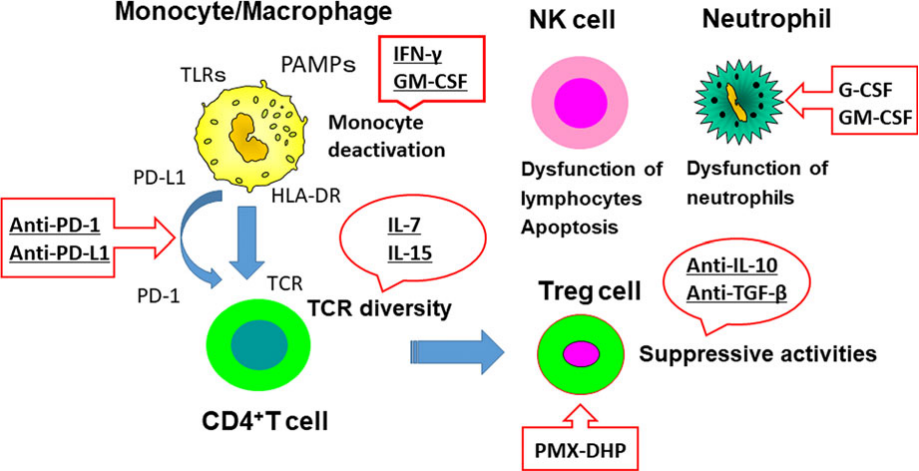
Immunostimulation therapies for sepsis. Targets of potential immunotherapeutic approaches include agents that block apoptosis, block negative costimulatory molecules, decrease the level of anti‐inflammatory cytokines, increase human leukocyte antigen (HLA)‐DR expression, and reactivate “exhausted” or anergic T cells. G‐CSF, granulocyte colony‐stimulating factor; GM‐CSF, granulocyte‐macrophage colony‐stimulating factor; IFN, interferon; IL, interleukin; NK, natural killer; PAMP, pathogen‐associated molecular patterns; PD‐1, programmed cell death‐1; PD‐L1, programmed cell death 1 ligand 1; PMX‐DHP, polymyxin B direct hemoperfusion; TCR, T cell receptor; TGF, transforming growth factor; TLR, Toll‐like receptor

Patients with sepsis have a relatively short‐lived hyper‐inflammatory phase; therefore, drugs targeting inflammation have only a narrow time‐frame to exert their effects. Immunosuppression in sepsis thus provides a novel understanding of the disorder as well as a new therapeutic approach.^9^ Therefore, investigators have attempted to stimulate innate and adaptive immune systems with IFN‐γ, granulocyte‐macrophage colony‐stimulating factor (GM‐CSF), or granulocyte colony‐stimulating factor G‐CSF (Figure 3). Although it is possible that immunostimulatory therapy exacerbates the hyper‐inflammatory phase of sepsis, clinical trials of IFN‐γ, a potent immunostimulatory agent, and GM‐CSF in patients with various systemic inflammatory states did not elicit unbridled inflammatory reactions. Most patients with sepsis are severely immunosuppressed. Treatment with GM‐CSF was predicted to reverse the dysfunction of dendritic cells and monocytes/macrophages.^41^ G‐CSF was used to increase the number of polymorphonuclear leukocytes in an effort to enhance pathogen clearance. However, a recent meta‐analysis carried out on GM‐CSF and G‐CSF studies failed to show survival benefit.^42^

In addition to GM‐CSF, G‐CSF and IFN‐γ, other immunoadjuvant therapies, such as IL‐7 and IL‐15, may be effective in augmenting the adaptive immune system and restoring immunity. The profound apoptosis‐induced deletion of lymphocytes in sepsis is one such attractive therapeutic target. To prevent the extensive apoptosis‐induced deletion of immune effector cells in patients with sepsis, one potential strategy is the use of anti‐apoptotic, immunostimulatory cytokines, such as IL‐7 and IL‐15; both agents have shown efficacy in models of sepsis. Inoue et al^43^ reported that IL‐15 treatment increased lymphocyte survival, decreased the apoptosis of NK cells, dendritic cells, and T cells, and increased IFN‐γ secretion. Giving IL‐15 may have additional benefits of increasing NK cell and dendritic cell survival, whereas IL‐7 is thought to be a cytokine that is targeted more toward T cells. IL‐7 is a multifunctional cytokine of the immune system that affects both T cells and B cells and induces the proliferation of naïve and memory T cells.^44^ Venet et al^45^ showed that IL‐7 treatment of isolated lymphocytes from septic patients restored T‐cell proliferation and IFN‐γ secretion.

A recent study provided insight into the molecular mechanisms that underlie immune depression following sustained inflammation. This study showed a crucial monocyte‐macrophage protein known as PD‐1, which is found in patients infected with the human immunodeficiency virus (HIV). PD‐1, which is a negative costimulatory molecule expressed on immune effector cells, is upregulated along with its cognate ligand PD‐L1 during chronic HIV infection. PD‐1 impairs immunity by inducing apoptosis, increasing the production of IL‐10, preventing T‐cell proliferation, and causing T cells to become nonresponsive.^11^ Thus, PD‐1 affects immunosuppression through its effect on IL‐10 expression. Common to most septic patients is the increased expression of PD‐1 on T cells during the progression from hyper‐inflammation to hypo‐inflammation. Recent studies showed that disruption of the PD‐1/PD‐L1 axis either by genetic deletion or by pharmacological manipulation improves survival in bacterial and fungal murine sepsis.^46^, ^47^ Boomer et al showed that PD‐1 expression was increased in CD4 and CD8 T cells, whereas PD‐L1 expression was increased in antigen‐presenting cells as well as in the spleen and lungs of septic patients. This indicates that the PD‐1/PD‐L1 axis is present and may be dysregulated in human sepsis.^11^, ^48^ In oncology, anti‐PD‐1 and anti‐PD‐L antibody therapy have been used successfully to treat various tumors.^49^ These data indicate that blocking the PD‐1/PD‐L1 axis is a promising target for restoring immune function in human sepsis.

### 3.2 Polymyxin B direct hemoperfusion therapy for septic immunoparalysis

**Figure 4:**
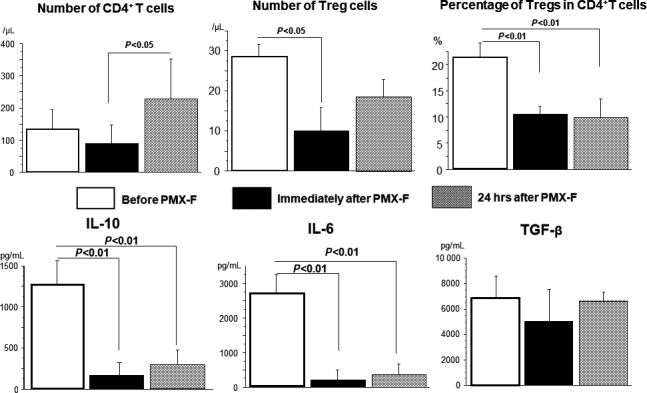
Changes in CD4^+^ T cells, Treg cells, and percentage of Treg cells among the CD4^+^ T‐cell population and serum cytokine levels before and after polymyxin B covalently immobilized on fibers (PMX‐F) therapy. Number of Treg cells was significantly decreased immediately after PMX‐F therapy, and the percentage of Treg cells in the CD4^+^ T‐cell population was significantly decreased immediately and 24 h after PMX‐F therapy compared with that before PMX‐F therapy. Both serum interleukin (IL)‐10 and IL‐6 levels were significantly decreased immediately and 24 h after PMX‐F therapy compared with before PMX‐F therapy (data from ref. (36)). TGF, transforming growth factor

Polymyxin B (PMX) has long been known to neutralize the various biological activities of endotoxins.^50^ In 1990, after the biocompatibility of sterilized PMX‐F (polymyxin B covalently immobilized on fibers) was demonstrated, the Critical Network Group in Japan obtained permission from the Japanese Ministry of Health and Welfare to clinically test hemoperfusion with PMX‐F, and subsequently reported that it was safe and effective for patients with septic shock.^51^, ^52^ The main objective of therapeutic apheresis is the removal of toxic substances, although the method can also be applied for immunomodulation. It was reported that the absorption of anandamide by polymyxin B might abolish the diverse negative effects of anandamide, such as hypotension, immunosuppression, and cytotoxicity,^53^ and that the reduction of serum cytokine levels^54^ or monocyte mRNA expression,^55^ and the proapoptotic activity of plasma^56^ from septic patients might contribute to the efficacy of hemoperfusion with PMX‐F. We previously reported that the expression of HLA‐DR surface antigen on monocytes is decreased in patients with septic shock, and that PMX‐F therapy is effective for increasing its expression.^26^ However, the molecular mechanism underlying these effects, and the potential of this treatment have not yet been fully analyzed. We found that there was an increase in the percentages of Treg cells in peripheral blood circulating CD4^+^ T cells from patients with sepsis, particularly those with septic shock, and that hemoperfusion with PMX‐F could remove Treg cells.^36^ Furthermore, the observed recovery of the number of CD4^+^ T cells and the decrease in the percentage of Treg cells in the CD4^+^ T‐cell population 24 hours after PMX‐F therapy may be useful prognostic immunological markers for patients with septic shock. Recently, we reported that there exists a significant positive correlation between serum IL‐10 levels and the percentage of Treg cells in the CD4^+^ T‐cell population in patients with postoperative infections, and that septic injury induced by cecal ligation and puncture increases the percentage of Treg cells among CD4^+^ T cells in the spleen, and there was a significant positive correlation between the percentage of Treg cells and serum IL‐10 or TGF‐β levels. Neutralization of IL‐10 or TGF‐β decreased the percentage of Treg cells among CD4^+^ T cells, restored the percentage of CD4^+^ T cells among spleen mononuclear cells, and improved survival rates in septic mice.^25^ We also found that PMX therapy directly reduces the number of Treg cells and improves the number of CD4^+^ T cells, which are key lymphocytes involved in maintaining immune responses under septic conditions (Figure 4). However, we cannot explain why hemoperfusion with PMX‐F reduces the number of Treg cells. Recently, Cappelli et al^57^ reported that polymyxin B positively modulates the depletion of Treg cells in the CD4^+^CD25^+^ population. Further research is hence necessary to confirm whether there is a causative relationship between the removal of specific lymphocytes and the beneficial effects of PMX‐F treatment in septic shock. The removal of Treg cells by hemoperfusion with PMX‐F might represent a novel strategy for inducing recovery from immunosuppressive conditions that arise during sepsis.

## 4 FUTURE ASPECTS

Immunotherapy is expected to be used in individual patients on the basis of specific laboratory or clinical findings. For example, a recent trial of GM‐CSF to treat sepsis tested the effect only on patients in whom monocyte HLA‐DR expression was significantly suppressed. Flow‐cytometry data regarding the expression levels of negative costimulatory molecules, such as PD‐1 and PD‐L1, on leukocytes may be useful as a guide for deciding on immunotherapies.

## DISCLOSURE

Conflicts of interest: Authors declare no conflicts of interest for this article.
